# The Southern Polar Front as a key to mesoplankton migratory behavior

**DOI:** 10.1038/s41598-020-70720-9

**Published:** 2020-08-20

**Authors:** Andrey Vedenin, Dmitry Kulagin, Eteri Musaeva, Alexander Vereshchaka

**Affiliations:** Shirshov Institute of Oceanology, Russian Academy of Sciences, Moscow, Russia 117997

**Keywords:** Food webs, Biooceanography, Community ecology, Ecosystem ecology, Marine biology

## Abstract

Diel and seasonal vertical migrations of zooplankton represent a widespread phenomenon occurring in marine and freshwater environments. Diel migrations are panoceanic, while seasonal migrations usually occur in temperate and polar areas. This paper describes differences in the diel and seasonal vertical migrations in the Drake Passage north and south of the Polar Front (PF). We analyzed material of 85 stations collected in spring of 2008 and 2010 (October–November) and in summer of 2010 and 2011 (January) within the 0–300 m depth range during various time of a day. At each station we sampled the upper mixed (UL), the middle (ML), and the deeper layers (DL) bounded by hydrological gradients. Diel migrations were significantly different south and north of the PF in terms of total abundance, biomass, diversity and individual taxa density. In both seasons, mesoplankton dielly migrated between the ML/DL and the UL north of the PF and between layers below 300 m and the DL and ML south of the PF. Deeper range of diel migrations south of the PF was coupled with a general mesoplankton descent in summer period compared to spring. Conversely, north of the PF, mesoplankton ascended to upper layers in summer, which was mirrored in lesser depths of diel migrations. The differences in the plankton distribution on both sides of the PF are likely associated with variations of vertical distribution of phytoplankton. Some abundant taxa such as *Aetideus* sp. and *Oithona plumifera* showed both common (nighttime ascend) and inverted (nighttime descend) vertical migrations depending on season and position related to the PF.

## Introduction

Diel and seasonal vertical migrations of zooplankton represent a well-known phenomenon observed for many species^1,2^. Adaptive significance of the migrations is mainly linked to feeding, predator-avoiding and/or reproductive behavior^2–9^.

The amplitude of diel vertical migrations ranges from a few to hundreds of meters^2,9,10^. Most migrating zooplankton species ascent at night (“normal” migration pattern); however, a reversed movement (nighttime descent, inverted migrations) is known for some species^2,5,9,11^. There are also more complicated migrations with a double ascent at sunset and after midnight with a short intermediate descent to deeper waters at midnight^7,8,12^. In addition, different stages of the same species may have different migration patterns, as shown for *Oithona* and *Oncaea* copepods^13^. Diel migrations of mesoplankton are believed to be caused by the predator evasion during daytime and by feeding during nighttime^3,14–16^.

Seasonal migrations of zooplankton to the surface waters for feeding and reproduction take place in temperate and polar areas, and are usually driven by phytoplankton bloom^16^. In autumn and winter, when the phytoplankton production is low, most species descend to deeper layers. In the Southern Ocean, phytoplankton spring and summer blooms occur in a rather thin upper mixed water layer < 100 m^17^, which results in an extensive ascent of the zooplankton to the upper waters with maximum densities in the upper 50 m^16^.

Both diel and seasonal migrations are greatly influenced by environmental factors including local hydrological conditions. In the Southern Ocean, these conditions are determined by the Antarctic Circumpolar Current (ACC). The ACC is known to be composed of several main jets and related hydrological fronts^18^, which act as boundaries for plankton communities^19^. The hydrological frontal system includes the Subtropical Front (STF), the Subantarctic Front (SAF), the Polar Front (PF), and the Southern Front (SF)^18^. Zones between the fronts represent biogeographic areas of the Southern Ocean, the PF marks one of the boundaries between the subantarctic and antarctic plankton assemblages^20–22^.

In the Southern Ocean, vertical diel migrations were studied for gelatinous plankton^19^, copepods^12,20,21^, and krill^23–25^. Until recently data on vertical migrations from different biogeographic areas were put in a common pool, which could mask local differences in migratory behavior. At the same time, we know that hydrological fronts greatly influence plankton biology (distribution, composition, biodiversity) and thus merit a deeper insight into their impact on vertical migrations^26,27^.

**Figure 1:**
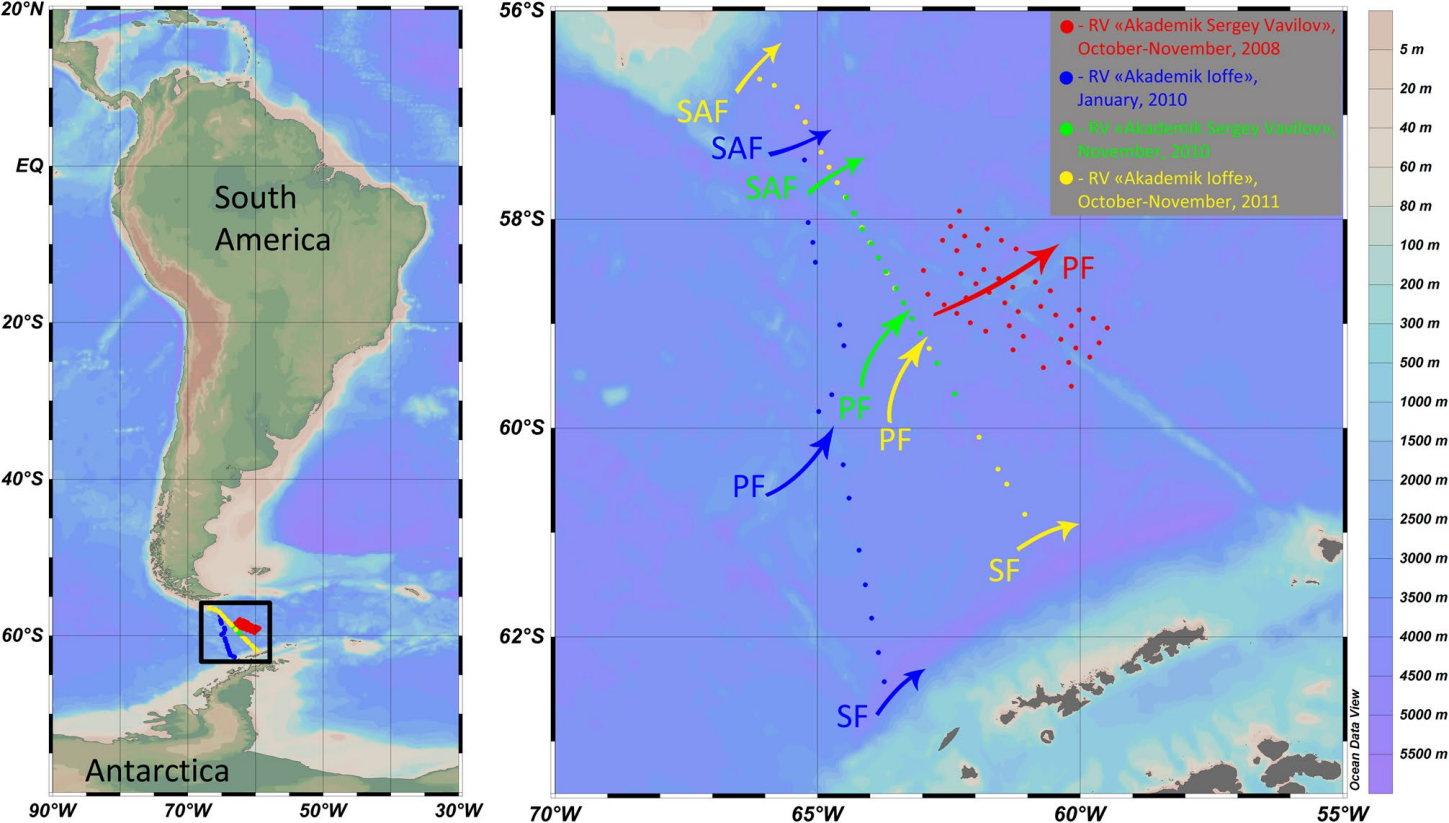
Study area with stations, arrows show running locations of the Subantarctic Front (SAF), the Polar Front (PF) and the Southern Front (SF). The map was generated using Ocean Data View v5.3.0 Software (https://odv.awi.de/).

In this study we focused on the impact of the PF on mesoplankton vertical migrations. The following hypothesis was tested: the PF influences seasonal and diel vertical migrations of the mesoplankton within the upper 300 m. We analyzed and compared diel dynamics of abundance, biomass and diversity of the mesoplankton on both sides of the PF on the basis of samples collected in the Drake Passage during four cruises at various time of day and at different depth ranges (Fig. 1). The sampled depth ranges included the upper mixed layer (UL, ~ 0–80 m) and the total epipelagic layer (TL, ~ 0–200 m) in spring 2008; the upper mixed layer (UL, ~ 0–80 m), the middle layer (ML, ~ 80–200 m) and the deeper layer (DL, ~ 200–300 m) in spring 2010–2011 and in summer 2010. Complete station list is presented in the Supplementary 1.

## Results

### Variability of community characteristics along the day

#### Spring samples

**Figure 2:**
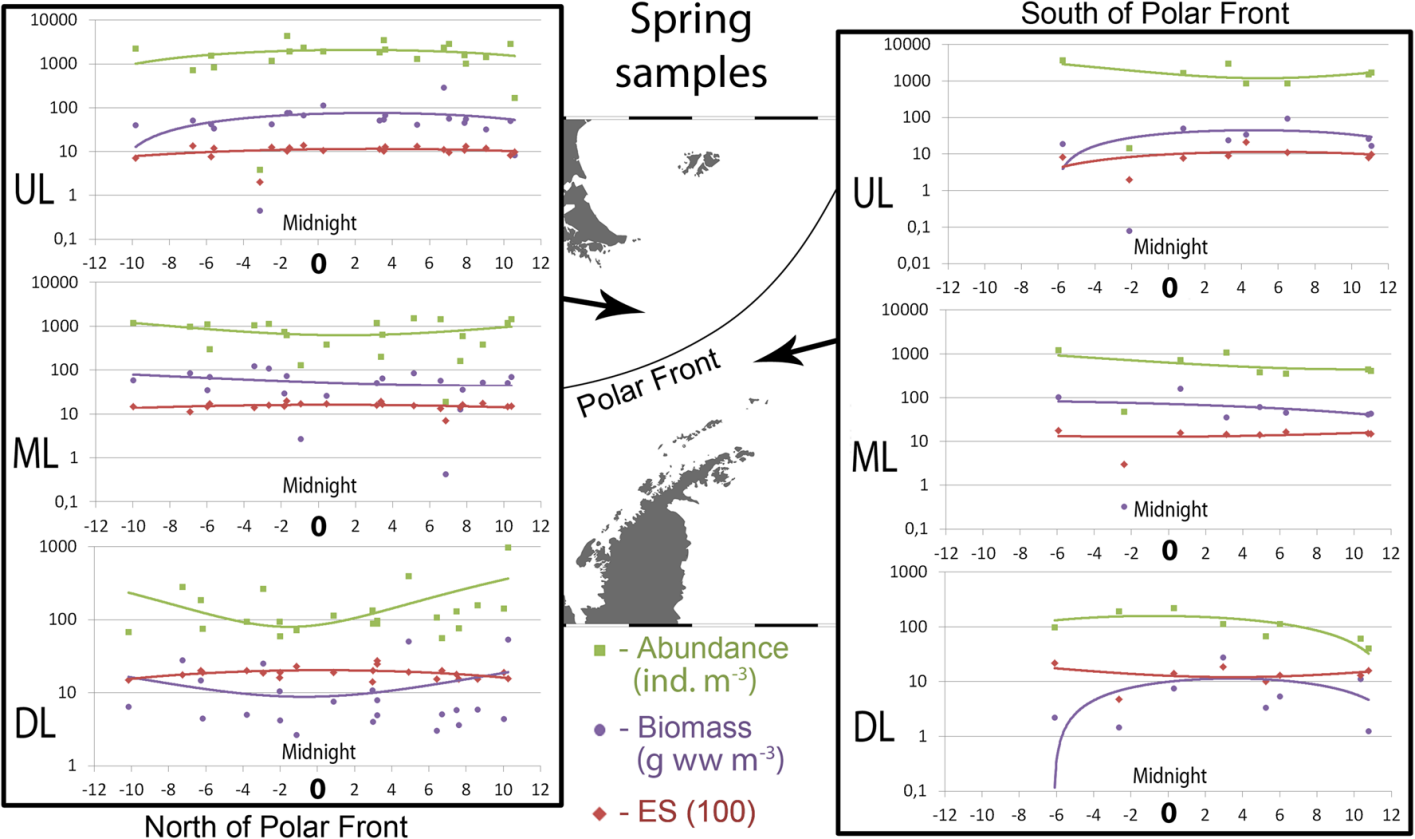
Values of abundance, biomass and ES (100) diversity values along the day in spring, 2010–2011. Horizontal axis shows daily position. Vertical axis is logarithmic and represents ind. m^−3^ for abundance, g ww m^−3^ for biomass; ES (100) is dimensionless. Left graphs show Upper, Middle and Deep layers north of Polar Front; Right graphs show Upper, Middle and Lower layers south of Polar Front. The map was generated using Ocean Data View v5.3.0 Software (https://odv.awi.de/).

**Figure 3:**
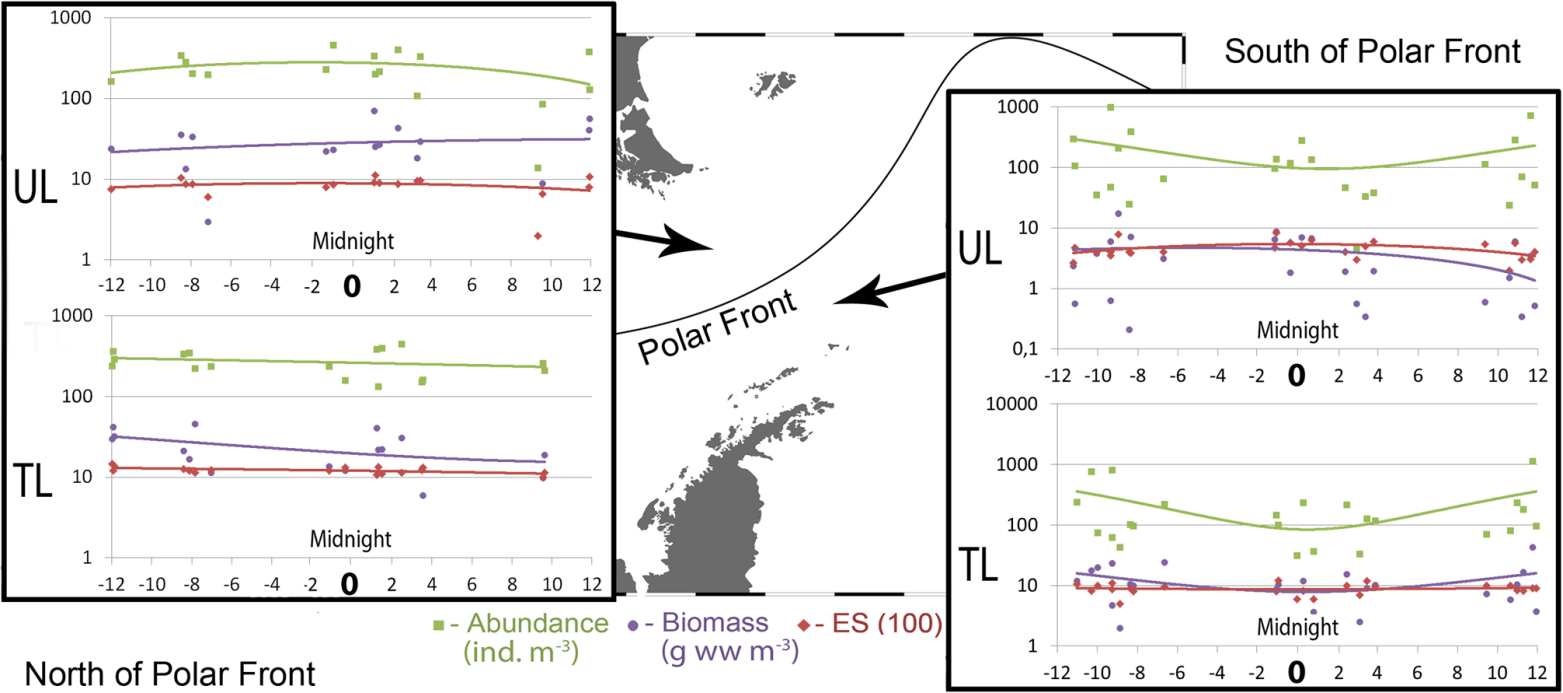
Values of abundance, biomass and ES (100) diversity values along the day in spring, 2008. Horizontal axis shows daily position. Vertical axis is logarithmic and represents ind. m^−3^ for abundance, g ww m^−3^ for biomass; ES (100) is dimensionless. Left graphs show Upper and Total epipelagic layers north of Polar Front; Right graphs show Upper and Total layers south of Polar Front. The map was generated using Ocean Data View v5.3.0 Software (https://odv.awi.de/).

North of the PF, abundance and biomass showed similar trends in the UL: values were maximal around the midnight with certain decrease towards the midday. South of the PF, trends of abundance in the UL were opposite (Fig. 2). Trends were similar in the ML on the both sides of the PF and opposite in the DL north and south of the PF. Both abundance and biomass increased around midnight in the DL south of the PF and decreased north of the PF (Fig. 2). Similar trends were recorded in 2008 (Fig. 3).

Diel dynamics of diversity were similar on both sides of the PF. Around midnight, diversity was maximal in the UL (Figs. 2, 3) and minimal or nearly constant in the ML and in the DL (Fig. 2). In 2008 diversity was nearly constant (Fig. 3).

Overall, the extrema of trends were located between the Time of Day values of -1 and + 2 north of the PF and between 0 and + 5 south of the PF (Figs. 2, 3).

#### Summer samples

**Figure 4:**
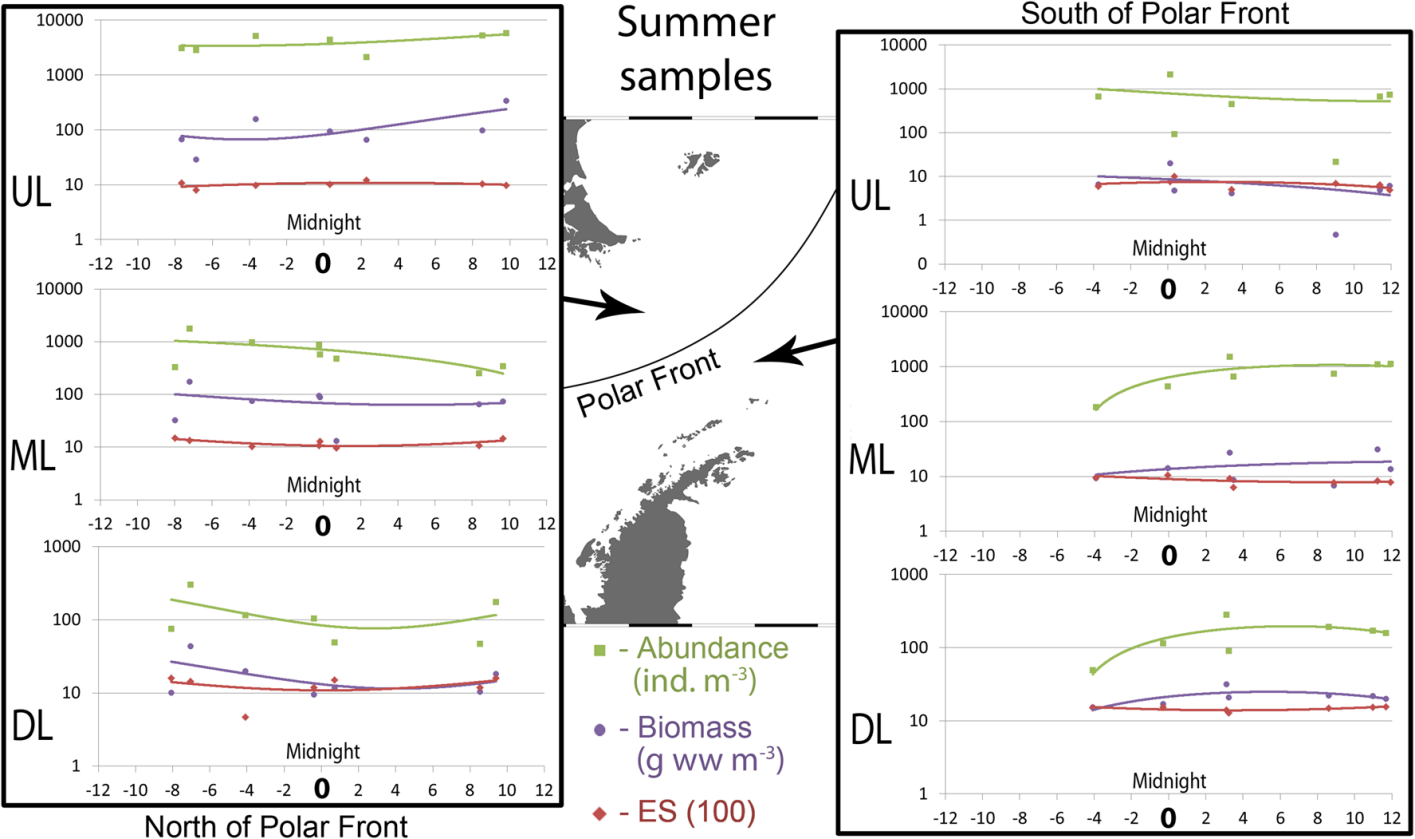
Values of abundance, biomass and ES (100) diversity values along the day in summer, 2010. Horizontal axis shows daily position. Vertical axis is logarithmic and represents ind. m^−3^ for abundance, g ww m^−3^ for biomass; ES (100) is dimensionless. Left graphs show Upper, Middle and Lower layers north of Polar Front; Right graphs show Upper, Middle and Lower layers south of Polar Front. The map was generated using Ocean Data View v5.3.0 Software (https://odv.awi.de/).

Trends in abundances and biomass were similar on both sides of the PF in the UL and ML and opposite in the DL: minimal around the midnight north of the PF and maximal around the midnight south of the PF (Fig. 4).

Diel dynamics of the diversity (species number and ES100) in summer were similar to those in spring with the midnight peak in upper layers and reverse pattern in middle and deeper layers (Fig. 4).

Extrema of polynomial trends were located in the same periods as in the spring time: between − 1 and + 2 north of the PF and between 0 and + 5 south of the PF (Fig. 4).

#### Daily position of sample: correlation

Despite visible trends (Figs. 2, 3, 4), only few integral parameters showed reliable Spearman ranked correlation with daily position (Table 1). Strong negative correlations were observed for the total abundance in the DL south of the PF in spring (*p* value 0.007). Positive correlation, although less significant, was recorded for the spring UL north of the PF in 2008 for abundance and in 2010–2011 for biomass (*p* values 0.08 and 0.04, respectively, Table 1). Diversity values showed *p *values < 0.05 in most layers during the spring season north of the PF. In summer, negative correlation of species number and daily position was observed (Table 1).

**Table 1:** Values of Spearman ranked correlation (R and *p* values) between Time of Day and integral community parameters and certain species for each layer, each season and each water zone.

Parameter	North of Polar Front						South of Polar Front					
	UL		TL				UL		TL			
	R	*p*	R	*p*			R	*p*	R	*p*		
**Spring (October–November, 2008)**												
**Abundance**	− 0.43	0.082	0.19	0.462			0.09	0.686	0.32	0.126		
**Biomass**	− 0.07	0.801	0.22	0.395			− 0.34	0.108	0.30	0.148		
**Species richness**	− 0.60	0.010	− 0.10	0.693			− 0.29	0.175	− 0.06	0.783		
**ES(100)**	− 0.29	0.265	0.19	0.468			− 0.53	0.008	0.19	0.368		
*Clausocalanus* sp.	− 0.86	7.45E–06	− 0.45	0.068			− 0.05	0.817	− 0.23	0.275		
*Ctenocalanus citer*	− 0.81	8.03E–05	0.21	0.428			− 0.77	0.000	0.30	0.153		
*Metridia lucens*	0.08	0.765	0.76	3E–04			− 0.47	0.020	0.64	0.001		
*Microcalanus pygmaeus*	− 0.16	0.535	0.48	0.051			0.11	0.621	0.50	0.013		
*Pleuromamma robusta*	− 0.69	0.002	− 0.73	0.001			− 0.29	0.175	− 0.70	1E–04		
*Rhincalanus gigas*	0.00	0.985	0.30	0.243			− 0.27	0.204	0.36	0.082		
*Scolecithricella minor*	− 0.55	0.021	0.56	0.020			− 0.08	0.712	0.31	0.145		
Heterorabdidae gen.sp.	–	–	− 0.04	0.871			–	–	0.45	0.026		
*Oithona* sp.	− 0.24	0.353	0.07	0.779			0.19	0.376	0.38	0.064		
*Oncaea* sp.	− 0.46	0.064	0.13	0.619			− 0.03	0.892	0.49	0.015		
Harpacticoida	− 0.40	0.112	− 0.52	0.032			− 0.16	0.460	0.24	0.254		
Copepoda nauplii	− 0.45	0.068	0.09	0.825			− 0.55	0.005	0.23	0.278		
Appendicularia	− 0.05	0.837	0.27	0.295			0.11	0.597	0.41	0.048		

	UL		ML		DL		UL		ML		DL	
	R	*p*	R	*p*	R	*P*	R	*P*	R	*p*	R	*P*
**Spring (October–November, 2010; 2011)**												
**Abundance**	− 0.17	0.475	0.28	0.223	0.23	0.299	0.19	0.665	− 0.05	0.935	− 0.88	0.007
**Biomass**	− 0.45	0.043	0.06	0.731	0.10	0.9245	− 0.07	0.840	− 0.09	0.840	− 0.29	0.462
**Species richness**	− 0.57	0.007	− 0.08	0.723	0.12	0.582	− 0.19	0.665	0.01	0.990	0.31	0.450
**ES(100)**	− 0.27	0.239	− 0.43	0.048	− 0.45	0.035	0.48	0.216	0.29	0.462	0.26	0.536
*Tomopteris* sp.	− 0.03	0.898	0.44	0.047	− 0.26	0.238	− 0.22	0.588	− 0.13	0.762	− 0.41	0.500
Bivalvia larvae	0.26	0.258	− 0.12	0.590	0.42	0.049	–	–	–	–	–	–
*Amallothrix dentipes*	–	–	0.46	0.038	0.06	0.796	–	–	–	–	− 0.25	0.750
*Candacia longimana*	− 0.37	0.099	–	–	− 0.17	0.459	–	–	–	–	–	–
*Candacia* sp.	− 0.30	0.182	0.44	0.043	0.13	0.565	–	–	− 0.31	0.500	− 0.25	0.750
*Calanus propinquus*	− 0.06	0.790	0.38	0.093	–	–	− 0.46	0.304	− 0.20	0.652	− 0.41	0.500
*Clausocalanus breviceps*	− 0.44	0.045	− 0.02	0.939	0.20	0.373	–	–	–	–	–	–
*Clausocalanus latiseps*	− 0.30	0.180	0.43	0.049	0.00	0.999	− 0.58	0.250	− 0.34	0.404	0.41	0.315
*Ctenocalanus citer*	− 0.31	0.169	0.30	0.186	0.32	0.147	− 0.21	0.582	− 0.10	0.840	− 0.64	0.083
*Euchaeta marina*	− 0.32	0.162	− 0.11	0.650	0.15	0.491	− 0.08	0.999	0.20	0.631	− 0.66	0.093
*Metridia lucens*	− 0.37	0.097	− 0.36	0.104	− 0.17	0.451	0.06	0.886	0.47	0.246	0.46	0.258
*Scolecithriella minor*	− 0.54	0.011	0.17	0.473	0.20	0.381	− 0.05	0.929	0.47	0.246	0.07	0.840
*Oithona similis*	− 0.09	0.712	0.37	0.100	0.35	0.113	− 0.10	0.840	0.10	0.840	− 0.86	0.007
Harpacticoida	− 0.37	0.096	0.11	0.633	− 0.12	0.605	− 0.16	0.750	0.08	0.999	0.25	0.750
Ostracoda	− 0.56	0.008	− 0.04	0.869	− 0.41	0.061	− 0.28	0.498	− 0.12	0.752	0.00	0.999
larvae Euphausiidae	− 0.40	0.070	0.10	0.672	− 0.10	0.662	0.762	0.037	− 0.31	0.462	− 0.14	0.703
Appendicularia	− 0.19	0.414	− 0.39	0.078	− 0.48	0.025	0.00	0.999	− 0.01	0.987	0.52	0.220
**Summer (January, 2010)**												
**Abundance**	0.61	0.139	− 0.52	0.197	0.07	0.840	0.04	0.906	0.32	0.444	0.11	0.783
**Biomass**	0.43	0.302	− 0.33	0.389	0.21	0.595	− 0.18	0.713	− 0.07	0.840	0.04	0.906
**Species richness**	− 0.32	0.444	0.04	0.946	− 0.78	0.048	− 0.71	0.086	− 0.27	0.552	0.47	0.291
**ES(100)**	− 0.11	0.782	0.40	0.327	0.32	0.444	− 0.64	0.110	− 0.46	0.302	0.57	0.167
Radiolaria gen.sp.	0.86	0.012	0.26	0.536	0.21	0.595	− 0.42	0.352	0.68	0.088	0.35	0.444
Globigerinidae gen.sp.	0.68	0.088	0.07	0.840	− 0.07	0.840	− 0.32	0.444	− 0.21	0.595	− 0.54	0.236
Polychaeta gen.sp.	− 0.57	0.167	0.26	0.536	0.75	0.066	− 0.35	0.438	− 0.19	0.719	0.25	0.595
Pteropoda gen.sp.	0.39	0.396	− 0.12	0.752	0.33	0.471	− 0.77	0.062	− 0.72	0.078	− 0.41	0.571
*Aetideus giesbrechti*	–	–	0.78	0.030	− 0.78	0.057	–	–	–	–	0.76	0.095
*Candacia cheirura*	0.41	0.571	0.74	0.060	–	–	–	–	–	–	–	–
*Calanoides acutus*	0.36	0.444	− 0.38	0.360	0.05	0.919	− 0.51	0.257	− 0.56	0.206	− 0.72	0.077
*Euchaeta marina*	− 0.79	0.067	0.00	0.999	− 0.82	0.034	–	–	− 0.29	0.498	0.29	0.498
*Pareuchaeta tonsa*	0.20	0.857	0.64	0.083	− 0.80	0.095	–	–	–	–	–	–
*Oncaea* sp.	− 0.40	0.571	0.29	0.462	− 0.80	0.095	–	–	− 0.09	0.905	0.33	0.471
Ostracoda	− 0.32	0.444	0.02	0.977	0.71	0.088	–	–	− 0.73	0.088	0.20	0.667
Salpa	–	–	–	–	–	–	0.00	0.999	0.80	0.095	− 0.20	0.857

Correlations with *p* values < 0.10 are marked with underline. *UL* upper layer, *TL* total layer, *ML* middle layer, *DL* deeper layer.

Abundance of many individual taxa was robustly correlated with daily position of samples. These were different larvae, Polychaeta, Pteropoda, Ostracoda etc. Dominant copepod species of the genera *Aetideus*, *Candacia*, *Calanus*, *Calanoides*, *Clausocalanus*, *Ctenocalanus*, *Euchaeta*, *Pareuchaeta*, *Rhincalanus*, *Scaphocalanus*, *Scolecithricella*, *Pleuromamma*, *Metridia, Oithona* and *Oncaea* also showed statistically significant diel dynamics (Table 1).

### Day–night comparison

#### Integral community characteristics

##### Spring

**Figure 5:**
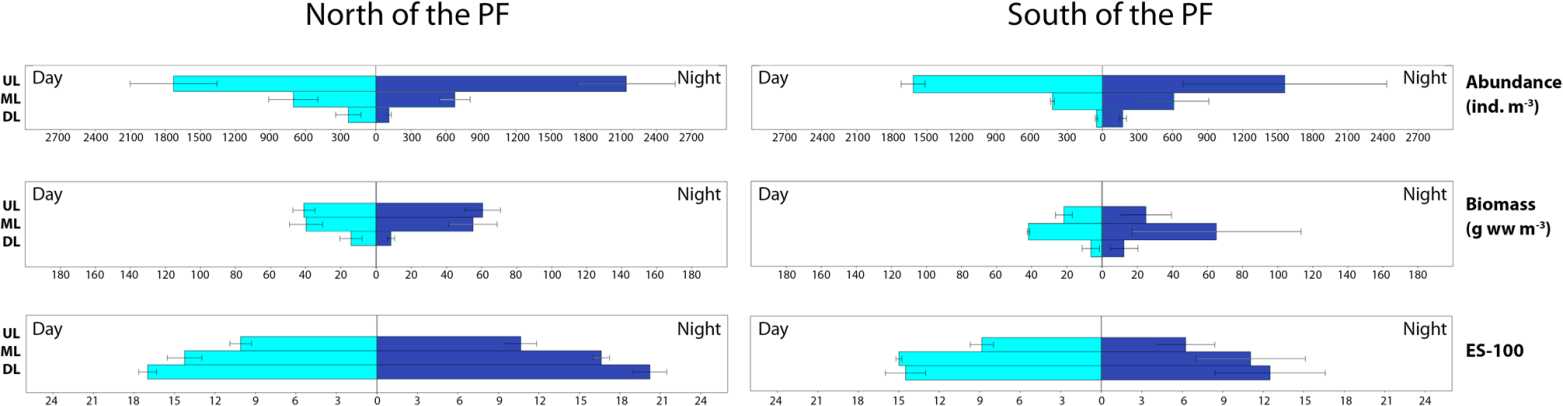
Abundance (ind. m^−3^), biomass (g ww m^−3^) and ES-100 values in different water layers in spring samples. Dark blue charts indicate night values; light blue charts indicate night values. Whiskers indicate standard error. *UL* upper layer, *ML* middle layer, *DL* deeper layer.

**Figure 6:**
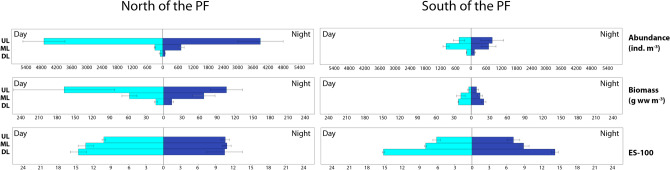
Abundance (ind. m^−3^), biomass (g ww m^−3^) and ES-100 values in different water layers in summer samples. Dark blue charts indicate night values; light blue charts indicate night values. Whiskers indicate standard error. *UL* upper layer, *ML* middle layer, *DL* deeper layer.

Comparison of averaged night/day ratios of total abundance, biomass and ES100 by layers is shown in Figs. 5 and 6. North of the PF, abundances in the UL were maximal at night (mean value—2,141 ind. m^−3^) and fell to 1731 ind. m^−3^ in the daytime; in the DL the dynamics were opposite (e.g. 110 vs. 235 ind. m^−3^). South of the PF, diel dynamics were different from that north of the PF: in the UL, the abundance maximum was observed during daytime (1619 ind. m^−3^ vs. 1561 ind. m^−3^); in the DL the mean night and day abundances were, respectively, 175 ind. m^−3^ and 51 ind. m^−3^ (Fig. 5).

Diel dynamics of biomass were similar to that of abundances on both sides of the PF and in all layers except that the highest biomass was observed in the ML instead of the UL south of the PF (Fig. 5). Diversity (ES100) values were generally higher in the ML and DL than in the UL on both sides of the PF, and the night/day differences were not significant (Fig. 5).

##### Summer

North of the PF, maximal zooplankton abundances were recorded in the UL and the values were higher than in spring (3,878 ind. m^−3^ at night and 4,717 ind. m^−3^ at day). In the DL abundances were lower than in spring (90 ind. m^−3^ at night and 99 ind. m^−3^ at day) (Fig. 6). South of the PF, distribution of abundances was different from that in spring: they were highest in the UL at night and in the ML at day (Fig. 6). North of the PF, biomass was maximal in the UL during daytime, while in the ML the night values were higher. South of the PF, biomass maximum was observed at night in the DL (Fig. 6). The diversity distribution south of the PF was also shifted to the DL, similar to the biomass dynamics (Fig. 6).

The overall differences in night and day distribution of integral parameters were lower in summer compared to spring (Table 2).

**Table 2:** Night/day ratios and *p* values of Student t-test of integral community parameters and certain species abundances.

Parameters	North of Polar Front						South of Polar Front					
	Night/day ratio			*p* value (T-test)			Night/day ratio			*p* value (T-test)		
	UL	TL		UL	TL		UL	TL		UL	TL	
**Spring (October–November, 2008)**												
**Abundance**	**2.06**	**1.42**		0.167	0.472		0.41	0.40		0.1704	0.170	
**Biomass**	**1.26**	0.90		0.422	0.700		**1.10**	0.61		0.8480	0.177	
**Species richness**	**1.25**	**1.03**		0.016	0.418		**1.05**	**1.06**		0.6268	0.325	
**ES (100)**	**1.15**	0.97		0.240	0.613		**1.30**	0.98		0.0654	0.810	
*Aetideus* sp.	–	0.95		–	0.426		–	**4.72**		–	0.030	
*Clausocalanus* sp.	**19.70**	**3.97**		0.042	0.154		0.50	**8.03**		0.6220	0.127	
*Ctenocalanus citer*	**7.27**	**1.77**		0.059	0.392		**11.66**	0.37		0.0075	0.222	
*Euchaeta marina*	–	–		–	–		–	–		–	–	
*Metridia lucens*	0.20	0.13		0.054	1E−04		**1.39**	0.09		0.6786	0.095	
*Pleuromamma robusta*	**∞**	**55.19**		0.058	0.001		**∞**	**∞**		0.2201	0.020	
*Scolecithricella minor*	**3.45**	0.60		0.084	0.030		**1.05**	0.45		0.9338	0.329	
*Oithona plumifera*	–	–		–	–		–	–		–	–	
*Oncaea* sp.	**16.60**	**1.45**		0.016	0.433		**1.65**	0.40		0.4636	0.183	
Harpacticoida gen.sp.	**14.61**	**∞**		0.213	0.015		**4.54**	0.45		0.1985	0.346	
Copepoda nauplii	**2.97**	0.91		0.143	0.871		**3.16**	**1.07**		0.0508	0.912	
Ostracoda gen.sp.	**1.23**	**1.60**		0.601	0.493		–	**2.32**		–	0.097	
Chaetognatha gen.sp.	–	–		–	–		–	–		–	–	
Appedicularia	**1.62**	**1.29**		0.488	0.745		0.44	0.13		0.3169	0.173	

	UL	ML	DL	UL	ML	DL	UL	ML	DL	UL	ML	DL
**Spring (October–November, 2010; 2011)**												
**Abundance**	**1.24**	0.95	0.47	0.489	0.894	0.222	0.96	**1.43**	**3.45**	0.962	0.663	0.061
**Biomass**	**1.48**	**1.39**	0.59	0.151	0.403	0.351	**1.15**	**1.55**	**1.98**	0.874	0.735	0.616
**Species richness**	**1.09**	**1.04**	0.94	0.526	0.825	0.508	0.98	0.80	0.95	0.978	0.707	0.907
**ES (100)**	**1.06**	**1.17**	**1.19**	0.712	0.093	0.049	0.71	0.74	0.86	0.432	0.508	0.737
*Aetideus* sp.	0.89	**3.16**	0.91	0.939	0.074	0.896	–	**∞**	**∞**	–	0.495	0.495
*Clausocalanus* sp.	**3.38**	0.52	0.77	0.112	0.144	0.652	**∞**	**20.45**	0.00	0.495	0.515	0.272
*Ctenocalanus citer*	**2.14**	0.64	0.31	0.188	0.388	0.116	**1.11**	**1.44**	**17.63**	0.901	0.717	0.437
*Euchaeta marina*	**∞**	**1.97**	0.24	0.229	0.475	0.323	–	0.17	**∞**	–	0.367	0.450
*Metridia lucens*	**39.13**	**1.72**	**1.15**	0.178	0.249	0.777	**8.87**	**1.51**	**1.31**	0.542	0.811	0.837
*Pleuromamma robusta*	–	**11.24**	**3.38**	–	0.247	0.273	–	**∞**	**1.25**	–	0.495	0.897
*Rhincalanus gigas*	**1.32**	**1.17**	0.10	0.580	0.768	0.274	**1.09**	0.67	0.76	0.938	0.510	0.664
*Scolecithricella minor*	**12.40**	0.84	0.53	0.038	0.720	0.285	**∞**	0.71	**1.32**	0.495	0.771	0.707
*Oithona plumifera*	**∞**	**1.36**	0.46	0.396	0.618	0.551	–	0.05	**1.75**	–	0.294	0.771
*Oncaea* sp.	**23.20**	**1.43**	0.43	0.353	0.350	0.356	0.36	**1.35**	**1.62**	0.474	0.72	0.762
Harpacticoida gen.sp.	**7.60**	0.00	**4.44**	0.205	0.271	0.414	**∞**	–	–	0.495	–	–
Copepoda nauplii	0.79	**1.12**	**1.74**	0.679	0.854	0.437	**1.67**	**4.29**	**2.11**	0.779	0.596	0.632
Ostracoda gen.sp.	**4.28**	**1.08**	**1.55**	0.014	0.781	0.365	**4.36**	**1.90**	**1.37**	0.596	0.649	0.638
Chaetognatha gen.sp.	**2.15**	**2.27**	0.70	0.229	0.010	0.292	0.47	**1.36**	**1.80**	0.562	0.776	0.469
Appedicularia	**1.05**	**25.01**	**∞**	0.945	0.145	0.031	**1.51**	**3.84**	0.33	0.822	0.611	0.495
**Summer (January, 2010)**												
**Abundance**	0.82	**2.35**	0.90	0.533	0.034	0.835	**1.76**	0.70	0.77	0.551	0.444	0.552
**Biomass**	0.63	**1.18**	**1.07**	0.530	0.687	0.839	**2.33**	0.86	0.99	0.327	0.787	0.978
**Species richness**	**1.07**	0.90	**1.13**	0.633	0.367	0.152	**1.19**	**1.03**	0.92	0.029	0.830	0.324
**ES (100)**	**1.04**	0.82	0.72	0.653	0.138	0.291	**1.17**	**1.12**	0.94	0.486	0.441	0.235
*Aetideus* sp.	–	0.08	**5.18**	–	0.002	0.015	–	–	0	–	–	0.064
*Clausocalanus* sp.	1.00	0.44	**1.29**	0.999	0.145	0.528	**19.50**	**5.01**	0.33	0.245	0.352	0.204
*Ctenocalanus citer*	–	–	–	–	–	–	**1.52**	**1.20**	0.82	0.661	0.872	0.862
*Euchaeta marina*	**∞**	0.62	**1.90**	6E–04	0.073	0.151	–	**1.64**	1.00	–	0.396	0.981
*Metridia lucens*	**∞**	0.46	0.48	0.374	0.284	0.268	–	**∞**	0.40	–	0.236	0.379
*Pleuromamma robusta*	**∞**	**∞**	**∞**	0.374	0.344	0.374	–	–	**∞**	–	–	0.437
*Rhincalanus gigas*	0.25	0.86	0.72	0.320	0.791	0.489	**∞**	0.83	0.83	0.437	0.823	0.183
*Scolecithricella minor*	0.00	–	–	0.374	–	–	**∞**	**2.49**	**1.54**	0.437	0.485	0.565
*Oithona plumifera*	0.02	0.59	**3.57**	0.338	0.325	0.032	–	**∞**	**1.65**	–	0.437	0.637
*Oncaea* sp.	0.01	0.89	0.40	0.319	0.843	0.212	**1.50**	**2.67**	**1.82**	0.809	0.555	0.650
Harpacticoida gen.sp.	**2.55**	0.23	0.82	0.323	0.089	0.894	**4.95**	0.78	0.00	0.516	0.867	0.286
Copepoda nauplii	0.43	0.82	**1.10**	0.383	0.790	0.928	**13.67**	**1.48**	**2.05**	0.457	0.604	0.594
Ostracoda gen.sp.	**1.51**	0.92	0.68	0.565	0.850	0.180	–	**∞**	0.44	–	0.211	0.331
Chaetognatha gen.sp.	0.27	0.95	**1.14**	0.209	0.881	0.753	–	**1.05**	0.98	–	0.944	0.954
Appedicularia	0.82	**4.98**	0.90	0.863	0.300	0.911	**3.81**	**1.56**	0.20	0.192	0.757	0.065

*p* values < 0.05 and corresponding night/day ratios are marked with underline. Parameters higher at night are bold. *UL* upper layer, *TL* total layer, *ML* middle layer, *DL* deeper layer.

#### Species distribution

**Figure 7:**
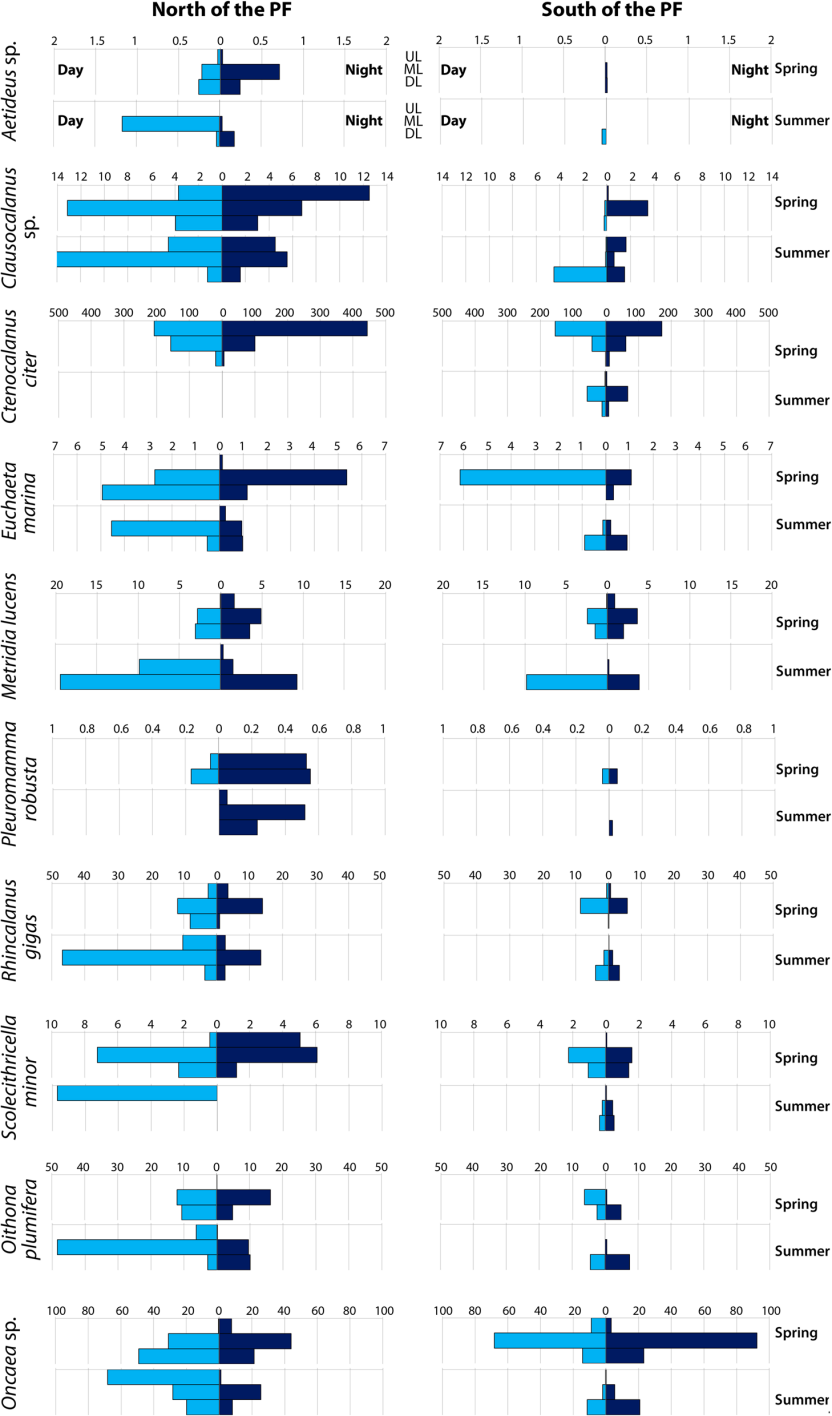
Mean abundance (ind. m^−3^) of several taxa in different water layers north and south of the Polar Front during spring and summer seasons. Dark blue bar charts indicate nighttime; light blue bar charts indicate daytime. *UL* upper layer, *ML* middle layer, *DL* deeper layer.

Distribution of some dominant taxa differed significantly in spring and summer samples (Table 2) including copepods, which showed correlation with diel position (see Section 3.1.3.). During the spring, some shallow-water species were clearly more abundant in the upper layers at night and in the deeper layers in the daytime (e.g. *Clausocalanus* sp.) (Fig. 7). In summer, the night ascent was less prominent or even absent among these species (Fig. 7). Mesopelagic species were more abundant at night in the ML and DL and nearly absent in the UL (e.g. *Aetideus* sp., *Euchaeta marina*, *Pleuromamma robusta*) (Fig. 7). Most taxa occurred deeper south of the PF than north of the PF, and deeper in summer than in spring (Table 2).

Copepods *Aetideus* spp., *Rhincalanus gigas*, *Oithona plumifera* and *Oncaea* sp. in summer migrated inversely (midnight decent—Table 3, Fig. 7). Correlation of abundances of *Aetideus* and *Oithona* species with abundances of possible predators revealed negative correlation values with hydromedusae, siphonophores and predatory copepods (*Euchaeta*, *Pareuchaeta* and *Heterorhabdus* genera) (Table 4).

**Table 3:** Mean number (N) and percentage (%) of species more abundant during nighttime or daytime in different layers, seasons and position relative to the PF, and their percentage.

	North of PF, UL				North of PF, TL		South of PF, UL				South of PF, TL	
	N	%			N	%	N	%			N	%
**Spring (October–November, 2008)**												
Night	12	60			11	48	8	50			10	40
Day	8	40			12	52	8	50			15	60

	North of PF, UL		North of PF, ML		North of PF, DL		South of PF, UL		South of PF, ML		South of PF, DL	
	N	%	N	%	N	%	N	%	N	%	N	%
**Spring (October–November, 2010; 2011)**												
Night	38	75	41	59	22	33	26	68	32	64	42	81
Day	13	25	28	41	44	67	12	32	18	36	10	19
**Summer (January, 2010)**												
Night	19	46	25	52	22	49	21	75	28	56	22	40
Day	22	54	23	48	23	51	7	25	22	44	33	60

*UL* upper layer, *ML* middle layer, *DL* deeper layer.

**Table 4:** Spearman ranked correlation (R and *p* values) of species with negative migration patterns with possible predators.

Predators	*Aetideus* sp.		*Aetideus armatus*		*Rhincalanus gigas*		*Oithona plumifera*		*Oncaea* sp.	
	R	*p*	R	*p*	R	*p*	R	*p*	R	*p*
**Spring (October–Novermber, 2010; 2011)**										
Hydromedusae	**− 0.25**	**0.0099**	0.38	5.05E−05	0.25	0.0085	**− 0.38**	**6.42E−05**	0.09	0.3601
Siphonophorae	0.33	0.0006	**− 0.32**	**0.0009**	0.19	0.0445	0.51	2.34E−08	0.27	0.0042
*Euchaeta marina*	0.60	6.18E−12	**− 0.39**	**2.99E−05**	0.39	3.21E−05	0.89	2.96E−38	0.49	8.70E−08
*Heterorhabdus austrinus*	0.17	0.0833	0.35	0.0002	0.00	0.9794	**− 0.25**	**0.0089**	0.15	0.1323
*Heterorhabdus papilliger*	0.52	1.13E−08	**− 0.31**	**0.0012**	0.13	0.1732	0.80	1.44E−24	0.43	3.47E−06
*Heterorhabdus spinifrons*	0.18	0.0675	− 0.08	0.4182	0.01	0.9329	0.17	0.0717	0.19	0.0496
*Pareuchaeta* sp.	− 0.01	0.9315	0.71	7.76E−18	0.14	0.1444	**− 0.45**	**1.07E−06**	0.33	0.0005
Chaetognatha	0.45	1.56E−06	0.13	0.1674	0.66	1.82E−14	0.43	3.59E−06	0.55	1.18E−09
Fish Larvae	− 0.11	0.2666	0.04	0.6610	0.19	0.0488	0.02	0.8663	− 0.16	0.1045
**Summer (January, 2010)**										
Hydromedusae	− 0.07	0.6170	− 0.04	0.7620	− 0.09	0.4959	− 0.14	0.2962	− 0.19	0.1523
Siphonophorae	− 0.15	0.2688	0.11	0.4221	− 0.06	0.6399	0.03	0.8508	0.32	0.0151
*Euchaeta marina*	0.64	5.21E−08	0.08	0.5580	0.71	3.29E−10	0.72	1.99E−10	0.54	9.83E−06
*Heterorhabdus papilliger*	− 0.07	0.6244	0.29	0.0246	0.14	0.2889	0.22	0.0990	0.10	0.4701
*Heterorhabdus spinifrons*	0.52	2.94E−05	0.06	0.6630	0.38	0.0034	0.49	9.00E−05	0.52	2.85E−05
*Paraeuchaeta exiqua*	− 0.07	0.6170	− 0.04	0.7620	0.04	0.7902	− 0.14	0.2962	0.10	0.4590
*Paraeuchaeta gracilis*	− 0.07	0.6170	0.43	0.0007	− 0.12	0.3899	− 0.14	0.2962	0.01	0.9294
*Paraeuchaeta langae*	− 0.07	0.6170	− 0.04	0.7620	− 0.04	0.7451	0.10	0.4435	0.18	0.1801
*Paraeuchaeta sarsi*	− 0.07	0.6170	− 0.04	0.7620	− 0.19	0.1523	− 0.04	0.7831	− 0.07	0.6150
*Paraeuchaeta tonsa*	0.55	8.96E−06	− 0.07	0.5820	0.40	0.0016	0.56	5.71E−06	0.53	1.65E−05
*Paraeuchaeta* sp.	− 0.07	0.6170	0.40	0.0020	− 0.15	0.2717	− 0.14	0.2962	− 0.06	0.6573
Chaetognatha	0.36	0.0050	0.08	0.5390	0.80	3.02E−14	0.50	6.06E−05	0.50	6.03E−05
Fish larvae	− 0.07	0.6170	− 0.04	0.7620	− 0.08	0.5740	− 0.14	0.2962	− 0.08	0.5342

Negative correlation pairs are marked with underline; negative correlation values with *p* values < 0.05 are marked with bold.

Overall, the UL was more enriched in taxa at night than in the daytime (46–75% vs. 25–54% of recorded taxa). The ML was still more enriched at night, but the night/day differences were less significant (52–64% at night vs 36–48% in the daytime). In the DL the proportions varied depending on the zone and/or the season (Table 3).

## Discussion

Vertical migrations of Subantactric and Antarctic zooplankton were previously studied in the Drake Passage and adjacent Southern Ocean both in diel and seasonal aspects^16,19–21,28,29^. One of the common ways to describe the magnitude of diel migrations is a night/day ratio of mesozooplankton characteristics^20,29,30^. As an example, the mean night/day ratios of the mesoplankton abundance and biomass were reported to be 1.06 and 1.17, respectively, in the upper 100 m of the Amundsen Sea^29^. In waters around South Georgia the night/day rations varied from 0.61 to 1.69 and from 0.73 to 1.45 for abundance and biomass, respectively^20^. The ratios were obtained from the station pools not divided by any of the hydrological fronts^20,29^. However, the fronts may drastically influence mesoplankton distribution^19–22^, so we hypothesized that the observed variations are linked to the position of main hydrological fronts. Indeed, in our dataset, the ratio depended on the position respective to the PF, varying in wide range from 0.47 north of the PF to 3.45 south of the PF (Table 2). Additionally to the mesoplankton abundance and biomass, the similar night/day differences were also demonstrated for the diversity values, which was never reported before. The ES-100 changes indicate that the vertical migration involves many mesoplankton species in a similar way (including the rare ones), as this index includes both number of species and the evenness of their abundances^31,32^.

The diel vertical migrations are thought to be triggered by the light intensity changes during the day^9,16,20,29,30^. The upward migrations were previously recorded around 7:00–9:00 p.m., with the following morning descent at 5:00–6:00 a.m.^16^. The difference in time is explained by changes in dusk and dawn time linked to different latitude or date^15,16,33^. In our samples, according to the distribution of integral parameters along the day, the nighttime extremum (maximum or minimum, depending on the PF position and the depth range) fell within the 11:00 p.m. and 3:00 a.m. north from the PF and within 1:00 a.m. and 4:00 a.m. south from the PF. The exact time of mesoplankton ascent and descent is hard to assess in our dataset.

The range and direction of diel vertical migrations in our samples depended not only on the position respective the PF (north or south of that) but also on the season (spring or summer). Seasonal changes in vertical migrations of zooplankton were regularly observed in temperate and polar seas^16,21,28,30,33–35^. The seasonal variation is often attributed to the overall decrease of diel light/dark cycles in summer^30,34,35^. In particular, the midnight sun period can stop any vertical movement of certain copepod species, as it was shown for *Calanus* species^35,36^. In winter, during the polar night, the mesoplankton migrates dielly despite the lack of obvious light/dark cycles^37^. In the Southern Ocean, an overall downward migration of zooplankton was observed in winter, as it was shown by summer/winter comparison^16,21,28^. Cisewski et al.^16^ distinguished two periods of migration patterns: from February to October (late summer to early spring), when the migrations are driven by the day/night rhythm; and from October to January, when the most of the zooplankton rises to the uppermost waters (< 50 m) for feeding and reproduction. Our samplings, taken in October–November (spring) and January (summer), therefore, fall into the second migration period described by^16^.

Differences between the spring and summer samples were significant and concerned integral community parameters and distribution of certain species. The seasonal shifts in mesoplankton vertical migrations depended on the position respective to the PF. Spring/summer vertical distribution of the abundance and biomass demonstrated the overall summer ascent north of the PF and summer descent south of the PF (Figs. 5, 6). In particular, north of the PF the mesoplankton was mainly concentrated in the upper layers by the summertime. South of the PF, the nighttime maxima of the abundance and biomass descended to the depth of ~ 150–300 m by summer (possibly even deeper, to the depths unsampled in this study) (Figs. 5, 6). Seasonal zooplankton migrations are linked to the food source distribution (i.e. primary production rate, often expressed in Chl *a* values) which concentrates in the upper mixed layer during spring and summer^16^. In the Drake Passage, the maximum surface chlorophyll values were observed around November–December north of the PF and in December-January south of the PF^38,39^. Concentration of the mesoplankton in the UL north of the PF in spring and summer is thus explainable (Figs. 5, 6). However, the summer mesoplankton descent from the most productive UL to the deeper layers south of the PF is unexpected. A possible explanation may be in vertical distribution of the phytoplankton: e.g., in the Eastern Atlantic Sector of the Southern Ocean Chl *a* was concentrated in the UL north of the PF, but evenly distributed within the upper 100 m south of the PF^40^. In the Drake Passage south from the PF, the Chl *a* vertical distribution demonstrated deep maxima below the UL (pers. comm. by Dr. A. Demidov, unpublished data). The dynamics of individual species distribution expectably demonstrated similar migration patterns as the integral community parameters. Most recognized taxa migrated normally with the nighttime surface ascent, confirming previous studies^2,5,16,21,22,28^. However, several taxa demonstrated the reverse migration pattern with the night descent to the deeper waters. Previously the negative pattern was shown for species of *Oithona similis* and *Oncaea curvata* copepods near the Antarctic coast (south off ACC)^13^.

Seasonal shifts (reflected mainly in summer ascent) were previously reported for several species of copepods in the Southern Ocean, including *Eucalanus longiceps*, *R. gigas*, *Neocalanus tonsus*, *Calanoides acutus*, *Calanus simillimus* and *Calanus propinquus*^21^. Our data confirmed the spring/summer shifts for most of the species with possible exception of *C. acutus*. *E. longiceps* and *N. tonsus* were not statistically represented in our samples. According to our data, *R. gigas, O. plumifera*, *Oncaea* sp. and *Aetideus* spp. copepods changed direction of their diel migrations from normal to inversed depending on season and position respective to the PF. Inversed migrations are generally explained by a possible avoidance of nocturnal predators with normal migration patterns^5,15,30^, which is in accordance with our dataset: *Aetideus* sp. and *O. plumifera* show certain negative correlations with predators, including cnidarians and Euchaetidae copepods. These predatory mesoplankton organisms are listed as consumers of copepods, possibly including *Oithona* species^41^. We suggest that the predators influence migratory behavior of the taxa and may switch direction of migrations on both sides of the Polar Front. The observed details of the vertical migrations show fine adaptive adjustments of taxa to local factors depending on season (spring or summer), hydrological setting (north or south of the PF), and predators.

**Figure 8:**
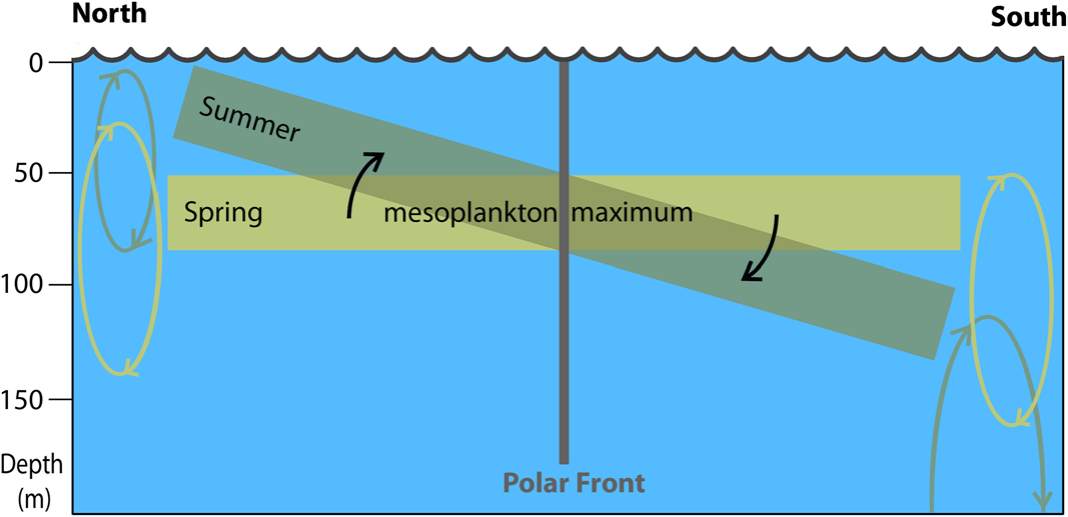
Scheme of spring-summer balance-like swing of mesoplankton maximum in the Drake Passage. Rectangles and elliptic arrows indicate position and ranges of diel migrations in spring (yellow) and summer (brown).

The PF has a great impact on seasonal and vertical migrations of mesoplankton. Vertical dynamics differ on both sides the PF in seasonal and diurnal aspects. North of the PF the mesoplankton concentrates in the UL both in spring and in summer, while south of the PF the mesoplankton concentrates in the UL in spring and descends in deeper layer in summer. In spring north of the PF, most of the taxa dielly migrate within the upper 300 m, ascending from the DL to the UL at night, while south of the PF vertical migrations encompass deeper layers from below 300 m (unsampled in this study) to the DL. In summer and north of the PF, migrations of mesoplankton are concentrated in the UL and large-scale diel migrations are insignificant, which mirrors feeding and reproduction in phytoplankton rich strata. Conversely, south of the PF the mesoplankton is concentrated below the UL: a possible result of an even vertical distribution of phytoplankton with no prominent surface maximum. The described trends of the seasonal and diel migrations are shown in a simplified scheme representing a balance-like swing of mesoplankton maxima on northern and southern sides of the PF (Fig. 8). Individual taxa such as *Aetideus* sp. and *O. plumifera* showed both common (nighttime ascend) and inverted (nighttime descend) vertical migrations depending on season and position related to the PF. According to^42^, the SF and the SAF act as more important boundaries for zooplankton communities than the PF. Although, we do not have a representative data set to compare the areas south from the SF and north from the SAF, we suggest that the differences in migration patterns in those areas may differ significantly from the described in this study.

## Material and methods

The material was based on four expeditions to the Drake Passage during early spring and summer. A total of 41 stations were sampled in October–November 2008 (RV “Akademik Sergey Vavilov”, 25-th expedition), 15 stations in January 2010 (RV “Akademik Ioffe”, 20-th expedition), 12 stations in November 2010 (RV “Akademik Sergey Vavilov”, 31-st expedition) and 17 stations in October–November 2011 (RV “Akademik Ioffe, 36-th expedition). All samples were taken using the Juday plankton net (mesh size of 0.18 mm, mouth area of 0.1 m^2^). In the expedition of 2008 two hauls at each station were taken, one in the upper mixed layer (UL, ~ 0–80 m), the other in the total epipelagic layer (TL ~ 0–200 m). In 2010 and 2011 expeditions three hauls were taken at each station in the upper 300-m active layer (i.e. in the whole epipelagic and the upper part of the mesopelagic). The three sampled strata were separated by vertical gradients of temperature and salinity. The uppermost mixed layer was well-defined and bounded from below by seasonal halo- and thermoclines; two deeper layers were separated from each other by the extrema of temperature and salinity profiles indicated by CTD-sensor at the same stations prior to biological sampling. Actual sampling depths ranged along the transect, the upper (UL) and middle layers (ML) typically represented the epipelagic, while the deep layer (DL) occurred mainly in the upper mesopelagic (Fig. 1, Supplementary 1). The net was equipped with the closing device; vertical towing speed was 1 m/sec. The hydrological setting was previously published by^43–45^.

Daily position of each sample was calculated individually. We used the local astronomical midnight as a zero-point, which was assessed on the basis of local time and coordinates (https://www.esrl.noaa.gov/gmd/grad/solcalc/sunrise.html). Daily position of each sample was further calculated as a difference between the astronomical midnight and sampling time and expressed in decimals (positive for a.m. time; negative for p.m. time—Supplementary 1).

Zooplankton samples were fixed with 4% formalin and later sorted in laboratory. All organisms were identified to the lowest possible taxonomical level. For each taxon, the numbers of specimens in the sample and individual sizes (length) were recorded with a precision of 0.1 mm. On the basis of this primary dataset, the individual weights, and biomass were calculated. Wet weight *w* of taxa represented by *i* specimens was estimated as *w* = Σ *(k* * *l*_*i*_^3^), where *l*_*i*_ is length of an individual specimen, *k* is a species-dependent coefficient; tables of these coefficients have been published by^46,47^. Abundance and biomass were normalized to ind. m^−3^ and g ww m^−3^, respectively, and presented in Supplementary 2.

Total abundance, biomass, species number and Hurlbert rarefaction index for 100 individuals (ES100) were used as integral community parameters. In order to avoid bias linked to unrepresentative sampling of larger organisms (jellyfishes, euphausiids) and Protozoa, we excluded these groups from abundance and biomass matrices. Correlation between Time of Day (daily position) and taxa abundances and integral community parameters was calculated using Spearman ranked correlation (modulus values of daily positions were used)^31^. To assess diel trends of different parameters, we plotted 2-nd order polynomial trendlines. Difference between day-time and night-time distribution was estimated using the night/day values ratios and the Student t-test. Night-time and day-time stations were identified as stations taken during ± 4 h from astronomical midnight and ± 4 h from astronomical midday, respectively. Horizontal box charts with standard errors were plotted for integral community parameters and for certain taxa to visualize differences between the depth layers and day/night distribution.

Statistical analyses were performed using Primer V6, Past 3 and Microsoft Excel 2010 software^48,49^.

## Supplementary information

Supplementary Legends.

Supplementary Table 1.

Supplementary Table 2.

## Data Availability

Supplementary information accompanies this paper as Supplementary 1 and 2.
