# Supplementary material for: The Southern Polar Front as a key to mesoplankton migratory behavior
Source: Sci Rep. 2020 Aug 20;10:14046. doi: 10.1038/s41598-020-70720-9 (PMC7441398; doi:10.1038/s41598-020-70720-9)
Supplement: Supplementary file 1 — Supplementary Legends. [file 41598_2020_70720_MOESM1_ESM.docx]

**The Southern Polar Front as a key to mesoplankton migratory behavior**

Andrey Vedenin^1^, Dmitriy Kulagin^1^, Eteri Musaeva^1^, Alexander Vereshchaka^1*^

^1^- Laboratory of plankton communities structure and dynamics, Shirshov Institute of Oceanology, Russian Academy of Sciences, Moscow, 117997, Russia

*- corresponding author, alv@ocean.ru

**Supplementary 1.**

Station data, including coordinates, date, sampling time and depth range, and Time of Day calculations for each sample.

UL – upper layer; TL – total layer; ML – middle layer; DL – deeper layer; PF – Polar Front.

**Supplementary 2.**

Abundance values for each species normalized to individuals per cubic meter.

The first excel sheet presents spring samples; the second excel sheet presents summer samples.
