# Supplementary material for: The Southern Polar Front as a key to mesoplankton migratory behavior
Source: Sci Rep. 2020 Aug 20;10:14046. doi: 10.1038/s41598-020-70720-9 (PMC7441398; doi:10.1038/s41598-020-70720-9)
Supplement: Supplementary file 2 — Supplementary Table 1. [file 41598_2020_70720_MOESM2_ESM.docx]

Supplementary 1. Station data and Time of Day calculations for each sample. UL – upper layer; TL – total layer; ML – middle layer; DL – deeper layer; PF – Polar Front

| **Cruise** | **Station** | **Latitude** | **Longitude** | **Date (dd-mm-yyyy)** | **Position relative to the PF** | **Depth range (m)** | | |  | **Sampling time, local (hh:mm)** | | | **Astronomical midnight, local time (hh:mm:ss)** | **Time of Day** | | |
| --- | --- | --- | --- | --- | --- | --- | --- | --- | --- | --- | --- | --- | --- | --- | --- | --- |
|  |  |  |  |  |  | **UL** |  | **TL** |  | **UL** |  | **TL** |  | **UL** |  | **TL** |
| **RV "Akademik Sergey Vavilov", 25-st expedition** | 2060 | -57,920 | -62,300 | 23.10.2008 | North | 0-85 |  | 0-210 |  | ~night |  | ~night | 0:53:29 | - |  | - |
|  | 2061 | -58,067 | -62,470 | 23.10.2008 | North | 0-75 |  | 0-205 |  | 12:55 |  | 13:02 | 0:54:10 | -11,99 |  | -11,87 |
|  | 2062 | -58,200 | -62,620 | 23.10.2008 | North | 0-80 |  | 0-185 |  | 17:00 |  | 17:04 | 0:54:46 | -7,91 |  | -7,85 |
|  | 2064 | -58,490 | -62,983 | 24.10.2008 | North | 0-85 |  | 0-165 |  | 2:05 |  | 2:12 | 0:52:09 | 1,21 |  | 1,33 |
|  | 2066 | -58,717 | -62,900 | 24.10.2008 | North | 0-55 |  | 0-255 |  | 10:30 |  | 10:33 | 0:55:45 | 9,57 |  | 9,62 |
|  | 2069 | -58,300 | -62,350 | 24.10.2008 | North | 0-60 |  | 0-220 |  | 23:40 |  | 23:45 | 0:53:33 | -1,23 |  | -1,14 |
|  | 2070 | -58,160 | -62,200 | 25.10.2008 | North | 0-85 |  | 0-236 |  | 3:15 |  | 3:22 | 0:52:50 | 2,37 |  | 2,49 |
|  | 2072 | -58,090 | -61,770 | 25.10.2008 | North | 0-70 |  | 0-196 |  | 12:45 |  | 12:55 | 0:51:07 | 11,90 |  | -11,94 |
|  | 2073 | -58,250 | -61,933 | 25.10.2008 | North | 0-90 |  | 0-230 |  | 16:37 |  | 16:44 | 0:51:46 | -8,25 |  | -8,13 |
|  | 2075 | -58,520 | -62,270 | 26.10.2008 | North | 0-90 |  | 0-200 |  | 0:25 |  | 0:33 | 0:53:00 | -0,88 |  | -0,33 |
|  | 2077 | -58,817 | -62,590 | 26.10.2008 | North | 0-60 |  | 0-180 |  | 10:15 |  | 10:28 | 0:54:17 | 9,35 |  | 9,56 |
|  | 2078 | -58,900 | -62,350 | 26.10.2008 | South | 0-82 |  | 0-205 |  | 13:40 |  | 14:35 | 0:53:20 | -11,22 |  | -10,31 |
|  | 2079 | -58,750 | -62,170 | 26.10.2008 | North | 0-65 |  | 0-200 |  | 17:45 |  | 17:50 | 0:52:36 | -7,13 |  | -7,04 |
|  | 2080 | -58,617 | -61,983 | 27.10.2008 | North | 0-75 |  | 0-226 |  | 2:18 |  | 2:22 | 0:51:46 | 1,44 |  | 1,50 |
|  | 2081 | -58,480 | -61,833 | 27.10.2008 | North | 0-65 |  | 0-200 |  | 4:20 |  | 4:25 | 0:51:10 | 3,48 |  | 3,56 |
|  | 2083 | -58,200 | -61,500 | 27.10.2008 | North | 0-85 |  | 0-230 |  | 12:46 |  | 12:52 | 0:49:50 | 11,94 |  | -11,96 |
|  | 2084 | -58,283 | -61,220 | 27.10.2008 | North | 0-100 |  | 0-205 |  | 16:20 |  | 16:25 | 0:48:43 | -8,48 |  | -8,40 |
|  | 2086 | -58,567 | -61,550 | 28.10.2008 | North | 0-65 |  | 0-220 |  | 2:00 |  | 2:05 | 0:49:57 | 1,17 |  | 1,25 |
|  | 2087 | -58,700 | -61,733 | 28.10.2008 | North | 0-65 |  | 0-200 |  | 4:10 |  | 4:20 | 0:50:41 | 3,32 |  | 3,49 |
|  | 2089 | -58,990 | -62,092 | 28.10.2008 | South | 0-54 |  | 0-220 |  | 12:30 |  | 12:37 | 0:52:07 | 11,63 |  | 11,75 |
|  | 2090 | -59,070 | -61,800 | 28.10.2008 | South | 0-50 |  | 0-190 |  | 15:30 |  | 15:35 | 0:50:57 | -9,35 |  | -9,27 |
|  | 2092 | -58,800 | -61,433 | 29.10.2008 | South | 0-100 |  | 0-200 |  | 1:30 |  | 1:38 | 0:49:25 | 0,68 |  | 0,81 |
|  | 2093 | -58,650 | -61,283 | 29.10.2008 | South | 0-55 |  | 0-210 |  | 3:44 |  | 3:55 | 0:48:49 | 2,92 |  | 3,10 |
|  | 2095 | -58,883 | -61,183 | 29.10.2008 | South | 0-65 |  | 0-195 |  | 12:00 |  | 12:06 | 0:48:25 | 11,19 |  | 11,29 |
|  | 2096 | -59,020 | -61,350 | 29.10.2008 | South | 0-50 |  | 0-190 |  | 14:45 |  | 14:50 | 0:49:05 | -10,07 |  | -9,98 |
|  | 2098 | -59,250 | -61,280 | 29.10.2008 | South | 0-45 |  | 0-175 |  | 23:40 |  | 23:45 | 0:48:48 | -1,15 |  | -1,06 |
|  | 2099 | -59,120 | -61,083 | 30.10.2008 | South | 0-81 |  | 0-190 |  | 4:35 |  | 4:42 | 0:47:57 | 3,78 |  | 3,90 |
|  | 2101 | -58,833 | -60,750 | 30.10.2008 | South | 0-65 |  | 0-190 |  | 11:20 |  | 11:25 | 0:46:37 | 10,56 |  | 10,64 |
|  | 2102 | -58,917 | -60,467 | 30.10.2008 | South | 0-75 |  | 0-190 |  | 15:24 |  | 15:30 | 0:45:29 | -9,36 |  | -9,26 |
|  | 2106 | -59,420 | -60,700 | 31.10.2008 | South | 0-60 |  | 0-185 |  | 16:20 |  | 16:25 | 0:46:23 | -8,44 |  | -8,36 |
|  | 2108 | -59,150 | -60,367 | 01.11.2008 | South | 0-60 |  | 0-185 |  | 0:22 |  | 0:44 | 0:45:01 | -0,38 |  | -0,02 |
|  | 2109 | -59,020 | -60,170 | 01.11.2008 | South | 0-40 |  | 0-170 |  | 4:07 |  | 4:12 | 0:44:13 | 3,38 |  | 3,46 |
|  | 2111 | -59,233 | -60,083 | 01.11.2008 | South | 0-55 |  | 0-180 |  | 12:34 |  | 12:40 | 0:43:53 | 11,84 |  | 11,94 |
|  | 2112 | -59,370 | -60,215 | 01.11.2008 | South | 0-65 |  | 0-190 |  | 16:23 |  | 16:32 | 0:44:24 | -8,36 |  | -8,21 |
|  | 2114 | -59,600 | -60,167 | 02.11.2008 | South | 0-60 |  | 0-180 |  | 0:56 |  | 1:01 | 0:44:12 | 0,20 |  | 0,28 |
|  | 2116 | -59,320 | -59,817 | 02.11.2008 | South | 0-60 |  | 0-185 |  | 10:04 |  | 10:10 | 0:42:48 | 9,35 |  | 9,45 |
|  | 2117 | -59,183 | -59,640 | 02.11.2008 | South | 0-80 |  | 0-159 |  | 13:34 |  | 13:41 | 0:42:05 | -11,13 |  | -11,02 |
|  | 2118 | -59,040 | -59,483 | 02.11.2008 | South | 0-55 |  | 0-180 |  | 17:58 |  | 18:03 | 0:41:28 | -6,72 |  | -6,64 |
|  | 2119 | -58,950 | -59,750 | 02.11.2008 | South | 0-70 |  | 0-200 |  | 23:38 |  | 23:45 | 0:42:32 | -1,08 |  | -0,96 |
|  | 2120 | -58,867 | -60,020 | 03.11.2008 | South | 0-50 |  | 0-180 |  | 3:05 |  | 3:11 | 0:43:36 | 2,36 |  | 2,46 |
|  | 2122 | -58,683 | -60,570 | 03.11.2008 | South | 0-80 |  | 0-190 |  | 11:37 |  | 11:45 | 0:45:48 | 10,85 |  | 10,99 |
|  | 2123 | -58,600 | -60,850 | 03.11.2008 | South | 0-100 |  | 0-200 |  | 15:48 |  | 15:55 | 0:46:55 | -8,98 |  | -8,87 |
|  |  |  |  |  |  | **UL** | **ML** | **DL** |  | **UL** | **ML** | **DL** |  | **UL** | **ML** | **DL** |
| **RV "Akademik Ioffe", 30-th expedition** | 2292 | -62,43 | -63,73 | 03.01.2010 | South | 0-45 | 45-167 | 162-302 |  | 3:45 | 3:35 | 3:25 | 0:19:15 | 3,43 | 3,26 | 3,10 |
|  | 2294 | -62,15 | -63,84 | 03.01.2010 | South | 0-40 | 39-160 | 161-300 |  | 12:25 | 12:15 | 12:00 | 0:19:41 | -11,91 | 11,92 | 11,67 |
|  | 2296 | -61,82 | -63,97 | 03.01.2010 | South | 0-40 | 40-160 | 163-300 |  | 20:35 | 20:25 | 20:15 | 0:20:13 | -3,75 | -3,92 | -4,09 |
|  | 2298 | -61,5 | -64,09 | 04.01.2010 | South | 0-40 | 40-175 | 175-300 |  | 4:00 | 3:50 | 3:35 | 0:21:09 | 0,35 | 3,48 | 3,23 |
|  | 2300 | -61,17 | -64,21 | 04.01.2010 | South | 0-40 | 40-180 | 181-300 |  | 11:45 | 11:35 | 11:20 | 0:21:38 | 11,39 | 11,22 | 10,97 |
|  | 2303 | -60,67 | -64,4 | 05.01.2010 | South | 0-40 | 40-200 | 202-300 |  | 0:30 | 0:20 | 0:05 | 0:22:50 | 0,12 | -0,05 | -0,30 |
|  | 2305 | -60,35 | -64,51 | 05.01.2010 | South | 0-40 | 39-171 | 171-305 |  | 9:25 | 9:15 | 9:00 | 0:23:17 | 9,03 | 8,86 | 8,61 |
|  | 2308 | -59,84 | -64,98 | 05.01.2010 | North | 0-40 | 40-190 | 200-315 |  | 20:45 | 20:35 | 20:20 | 0:25:10 | -3,67 | -3,84 | -4,09 |
|  | 2309 | -59,68 | -64,73 | 06.01.2010 | North | - | 40-180 | 180-300 |  | 0:10 | 0:10 | 0:00 | 0:24:36 | -0,24 | -0,24 | -0,41 |
|  | 2311 | -59,21 | -64,5 | 06.01.2010 | North | 0-55 | 48-204 | 204-310 |  | 8:55 | 8:45 | 8:55 | 0:23:41 | 8,52 | 8,36 | 8,52 |
|  | 2313 | -59,01 | -64,58 | 06.01.2010 | North | 0-55 | 55-200 | 202-300 |  | 16:45 | 16:25 | 16:20 | 0:24:00 | -7,65 | -7,98 | -8,07 |
|  | 2315 | -58,41 | -65,04 | 07.01.2010 | North | 0-50 | 50-240 | 303-410 |  | 0:45 | 0:15 | 0:05 | 0:26:17 | 0,31 | -0,19 | -0,35 |
|  | 2317 | -58,22 | -65,09 | 07.01.2010 | North | 0-70 | 70-210 | 212-300 |  | 10:15 | 10:05 | 9:50 | 0:26:29 | 9,81 | 9,64 | 9,39 |
|  | 2319 | -58,03 | -65,18 | 07.01.2010 | North | 0-50 | 50-200 | 202-300 |  | 17:35 | 17:15 | 17:25 | 0:26:51 | -6,86 | -7,20 | -7,03 |
|  | 2321 | -57,43 | -65,25 | 08.01.2010 | North | 0-70 | 70-150 | 160-320 |  | 2:45 | 1:50 | 1:10 | 0:27:33 | 2,29 | 0,71 | 0,71 |
| **RV "Akademik Sergey Vavilov", 31-st expedition** | 2259 | -59,67 | -62,39 | 07.11.2010 | South | 0-45 | 45-173 | 170-300 |  | 2:10 | 2:00 | 1:50 | 23:53:11 | 2,28 | 2,11 | 1,95 |
|  | 2261 | -59,38 | -62,72 | 07.11.2010 | South | 0-50 | 50-180 | 180-300 |  | 10:00 | 9:50 | 9:40 | 23:54:30 | 10,09 | 9,93 | 9,76 |
|  | 2263 | -59,09 | -63,04 | 07.11.2010 | South | 0-50 | 50-190 | 190-300 |  | 17:10 | 17:00 | 16:50 | 23:55:47 | -6,76 | -6,93 | -7,10 |
|  | 2264 | -58,95 | -63,20 | 07.11.2010 | North | 0-130 | 130-235 | 235-300 |  | 22:10 | 22:00 | 21:50 | 23:56:25 | -1,77 | -1,94 | -2,11 |
|  | 2265 | -58,80 | -63,36 | 08.11.2010 | North | 0-60 | 60-180 | 180-300 |  | 2:30 | 2:20 | 2:10 | 23:57:07 | 2,55 | 2,38 | 2,21 |
|  | 2266 | -58,66 | -63,52 | 08.11.2010 | North | 0-56 | 55-200 | 200-300 |  | 6:00 | 5:50 | 5:40 | 23:57:46 | 6,04 | 5,87 | 5,70 |
|  | 2267 | -58,51 | -63,69 | 08.11.2010 | North | 0-70 | 70-220 | 220-300 |  | 9:20 | 9:10 | 9:00 | 23:58:26 | 9,36 | 9,19 | 9,03 |
|  | 2268 | -58,37 | -63,84 | 08.11.2010 | North | 0-50 | 50-200 | 200-300 |  | 13:10 | 13:00 | 12:50 | 23:59:02 | -10,82 | -10,98 | -11,15 |
|  | 2269 | -58,23 | -63,99 | 08.11.2010 | North | 0-50 | 50-200 | 200-300 |  | 17:15 | 17:00 | 16:50 | 23:59:38 | -6,74 | -6,99 | -7,16 |
|  | 2270 | -58,09 | -64,15 | 08.11.2010 | North | 0-50 | 50-200 | 200-300 |  | 21:20 | 21:10 | 21:00 | 0:00:17 | -2,67 | -2,84 | -3,00 |
|  | 2271 | -57,94 | -64,30 | 09.11.2010 | North | 0-55 | 55-160 | 160-300 |  | 2:20 | 2:10 | 2:00 | 0:00:57 | 2,32 | 2,15 | 1,98 |
|  | 2272 | -57,79 | -64,46 | 09.11.2010 | North | 0-100 | 110-218 | 210-319 |  | 6:55 | 6:40 | 6:30 | 0:01:36 | 6,89 | 6,64 | 6,47 |
| **RV "Akademik Ioffe", 36-th expedition** | 2574 | -60,8250 | -61,0558 | 29.10.2011 | South | 0-88 | 88-156 | 156-300 |  | 19:40 | 19:25 | 19:10 | 23:47:57 | -4,13 | -4,38 | -4,63 |
|  | 2576 | -60,5385 | -61,3955 | 30.10.2011 | South | 0-100 | 98-200 | 200-300 |  | 4:20 | 4:10 | 3:50 | 23:49:15 | 4,51 | 4,35 | 4,01 |
|  | 2577 | -60,3933 | -61,5622 | 30.10.2011 | South | 0-70 | 70-187 | 187-300 |  | 8:45 | 8:35 | 8:10 | 23:49:55 | 8,92 | 8,75 | 8,33 |
|  | 2579 | -60,0862 | -61,9273 | 30.10.2011 | South | 0-75 | 80-223 | 210-300 |  | 22:40 | 22:30 | 22:10 | 23:51:22 | -1,19 | -1,36 | -1,69 |
|  | 2585 | -59,2362 | -62,8777 | 01.11.2011 | South | 0-77 | 73-203 | 190-300 |  | 2:10 | 2:50 | 3:10 | 23:55:05 | 2,25 | 2,92 | 3,25 |
|  | 2589 | -58,6590 | -63,5252 | 01.11.2011 | North | 0-65 | 60-217 | 212-300 |  | 18:50 | 18:30 | 18:10 | 23:57:40 | -5,13 | -5,46 | -5,79 |
|  | 2590 | -58,5143 | -63,6822 | 01.11.2011 | North | 0-89 | 90-189 | 191-331 |  | 22:15 | 22:25 | 22:50 | 23:58:18 | -1,72 | -1,56 | -1,14 |
|  | 2592 | -58,2258 | -63,9982 | 02.11.2011 | North | 0-90 | 90-198 | 105-300 |  | 5:45 | 5:35 | 5:25 | 23:59:32 | 5,76 | 5,59 | 5,42 |
|  | 2593 | -58,0802 | -64,1520 | 02.11.2011 | North | 0-69 | 69-158 | 158-350 |  | 9:35 | 9:25 | 9:15 | 0:00:09 | 9,58 | 9,41 | 9,25 |
|  | 2595 | -57,7897 | -64,4630 | 02.11.2011 | North | 0-81 | 90-287 | 265-346 |  | 17:25 | 17:10 | 16:45 | 0:01:23 | -6,61 | -6,86 | -7,27 |
|  | 2596 | -57,6470 | -64,6240 | 02.11.2011 | North | 0-80 | 80-195 | 192-300 |  | 21:30 | 21:20 | 21:00 | 0:02:02 | -2,53 | -2,70 | -3,03 |
|  | 2597 | -57,5023 | -64,7797 | 03.11.2011 | North | 0-85 | 75-215 | 315-300 |  | 2:40 | 2:30 | 2:00 | 0:02:39 | 2,62 | 2,46 | 1,96 |
|  | 2598 | -57,3577 | -64,9300 | 03.11.2011 | North | 0-74 | 69-210 | 223-311 |  | 7:00 | 6:50 | 6:40 | 0:03:15 | 6,95 | 6,78 | 6,61 |
|  | 2600 | -57,0680 | -65,2348 | 03.11.2011 | North | 0-77 | 77-145 | 145-300 |  | 16:20 | 16:10 | 15:50 | 0:04:28 | -7,74 | -7,91 | -8,24 |
|  | 2601 | -56,9235 | -65,3868 | 03.11.2011 | North | 0-83 | 86-200 | 200-305 |  | 20:35 | 20:25 | 20:10 | 0:05:04 | -3,50 | -3,67 | -3,92 |
|  | 2603 | -56,7182 | -65,8222 | 04.11.2011 | North | 0-85 | 80-164 | 164-300 |  | 4:30 | 4:20 | 4:05 | 0:10:49 | 4,32 | 4,15 | 3,90 |
|  | 2604 | -56,6567 | -66,1067 | 04.11.2011 | North | 0-111 | 111-218 | 218-300 |  | 8:10 | 8:00 | 7:45 | 0:07:57 | 8,03 | 7,87 | 7,62 |
